# Nanoparticles formation mechanisms through the spark erosion of alloys in cryogenic liquids

**DOI:** 10.1186/s11671-015-1212-9

**Published:** 2015-12-29

**Authors:** Gennady Monastyrsky

**Affiliations:** National Technical University of Ukraine “KPI”, 37, Peremogy Avenue, UA-03506 Kyiv, Ukraine

**Keywords:** Nanoparticles of alloys, Nanopowders, Spark erosion method, Shape memory alloys, Glass-forming Zr-based alloys, Martensite, TEM, HRTEM, 81. Materials science, 61.46.Df Structure of nanocrystals and nanoparticles, 1.07.Wx Nanopowders

## Abstract

Mechanisms of the formation of nanoparticles of some B2 shape memory intermetallic compounds, glass-forming Zr-based alloy, and pure Ti obtained by spark erosion method in liquid nitrogen and argon are considered. One of peculiarity is a foam-like structure, which covers the surface of micron-sized particles that appear during spark erosion. Such morphology is related to the nanosized particles gathered in agglomerates. Detailed examination of those particles allows proposed several mechanisms of their formation. The mechanisms explains two kinds of nanosized particles: particles of several tens and even hundreds of nanometers are formed due to explosion of molten droplets while the smaller particles having in turn a different structure and morphology are formed as a result of condensation of evaporated constituents under different conditions. The latter have the composition usually different from the target composition while the composition of the former is very close to the target (master alloy) composition.

## Background

Shape memory materials (Ti-Ni-based, Ni-Mn-Ga, Ni-Al, Cu-Al-Ni) and high-temperature shape memory materials (Ti-Ni-Hf, Ti-Ni-Zr) have attractive perspectives for practical application with the use of the nanostructures of various kinds in particularly. Their operation is closely connected with the fundamental problem of martensitic transformation in nanoscale objects like nanoparticles. Unfortunately, there is no universal method to obtain nanosized particles from the *pre-alloying materials with target composition*—the mandatory condition for their practical using.

The well-known method of gas or liquid atomization [1–3] is not suitable for the production of a significant amount of fine powder with particle sizes less than several microns. The yield of powder submicron- and nanosized alloys particles is considerably higher in mechanical alloying method [3–6]. The drawback of this method is that there is a significant level of contaminants or additional phases that appear in the powder, which is prepared from elemental materials. The advantages of the wire explosion method [7, 8], which yields particularly dispersed powders, are often frustrated by the high cost of equipment, energy consumption, and the small number of the powder obtained. Chemical synthesis approach successfully used for Au-Cd nanoparticles production [9] is heuristic by definition. In this work, we will focus on spark erosion method. The method allows obtaining alloy powder with a given chemical composition in a wide range of sizes, with a relatively low level of contaminants [10–32]. One of the features of this method is the foam-like structures, which coat the surface of micron-sized particles that appear during spark erosion [27–32].

Mostly, the interest given to the spark erosion powder is caused by the micron-sized particles, the finest of them are removed by washing. At the same time, the detailed examination of the structure of such particles can help to highlight the mechanism of spark erosion powder formation mechanism as was shown in [27, 31, 32]. The goal of this article is to classify the experimental results, which are able to clarify the possible mechanisms of nanoparticle formation.

## Methods

**Fig. 1:**
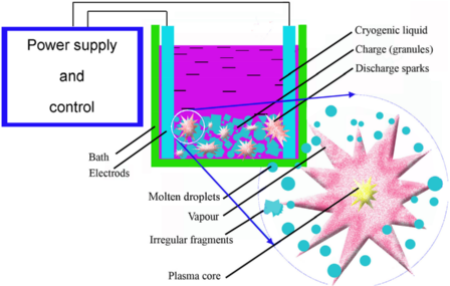
Principal scheme of the spark erosion working chamber. A similar chamber was used for the powder elaboration [26]. *Round inset* shows the formation during the spark discharge between the granules (1) the molten droplets, (2) the evaporated constituents of material, and (3) the irregular broken fragments of material pieces

The general principle of spark erosion processing was described in details in [10, 12, 25, 26]. It involves the application of a heavy current between two electrodes and a lot of small pieces sized 3–5 mm prepared from the pre-alloyed material, both being immersed in a dielectric coolant inside a container (liquid argon or liquid nitrogen). The key idea of the method is to apply the heavy electrical current of an electric discharge with a duration of 5–100 μs between the electrodes. Many sparks (arcs) simultaneously appear between the granules of the materials forming many conductive channels between the electrodes. When the spark collapses, molten droplets are ejected from these boiling regions with subsequent quenching in situ into coolant liquid (Fig. 1). Then, the powders are kept in a vessel with cryogenic liquid during the day until all liquid evaporated followed by passivation in hexane in order to prevent eventual explosion of the finest particles of the powders.

Pure Ti as well as Ni_49.0_Mn_28.5_Ga_22.5_, Cu_67.9_Al_27.5_Ni_3.8_Ti_0.5_Cr_0.3_, Ni_61.9_Al_38.1_, Ni_49_Cu_1_Ti_47_Zr_3_, Ni_49.9_Ti_40.3_Zr_0.3_Cu_0.1_Hf_9.4_, and Zr_57_Cu_20_Al_10_Ni_8_Ti_5_ alloys were used for powder preparation. Ni-Mn-Ga, Ti-Ni-Zr, and Ti-Ni-Hf alloys were prepared by induction melting while Ni-Al and Zr-Cu-Al-Ni-Ti by arc melting. Ti-Ni-based alloys were subjected to hot rolling followed by homogenization before spark erosion processing while the other ones were subjected only homogenization because of their brittleness. More details of the alloy and spark erosion powder production can be found in [27–32].

X-ray diffraction (XRD) and scanning electron microscopy (SEM) studies were carried out to reveal the crystal structure and morphology of the powders as a whole. The morphology, composition, and fine structure of the nanosized particles were investigated by different methods of analytical transmission electron microscopy (TEM), including high-resolution transmission electron microscopy (HRTEM), energy-filtered transmission electron microscopy (EFTEM), and energy-dispersive X-ray spectroscopy (EDX) analysis. For the sample preparation, the finest fraction of powders was mixed with ethanol; the mixture was dropped onto a copper grid covered by a carbon film. After evaporation of the ethanol, the sample was placed in the column of the microscope. A detailed description of the methods of structural investigations is provided in [27, 28, 31, 32].

## Results and Discussion

### Morphology of Spark Erosion Powders

**Fig. 2:**
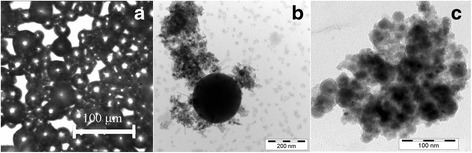
Different morphologies of the spark erosion powders particles. **a** The micron-sized Ni_49.0_Mn_28.5_Ga_22.5_ spherical particles solidified during the quenching in situ into liquid argon. **b** Cu_67.9_Al_27.5_Ni_3.8_Ti_0.5_Cr_0.3_ and **c** Zr_57_Cu_20_Al_10_Ni_8_Ti_5_ nanosized particles

It is considered that there are three main mechanisms of the powder particle formation: (a) mechanical breaking, (b) quenching from a liquid state, and (c) condensation from vapor states [29]. The morphology of the powder particles indirectly proves the mechanisms of the powder particle formation [28]. The shape of the largest particles (more than 30 ÷ 50 μm depending on the alloy) is mostly irregular confirming its origin due to mechanical shocks. The particles with dimensions less than 30–50 μm have a spherical shape (Fig. 2a). The part of such particles in the powder is close to 70 ÷ 90 % dependently on the alloys [27, 29, 31]. They are formed due to rapid quenching into coolant liquid. Hereinafter, we are concentrating on the particle sizes of several tens of nanometers (sometimes hundreds as shown on Fig. 2b). Most of such kind of particles are gathered in agglomerates (Fig. 2c).

**Fig. 3:**
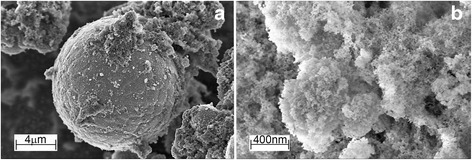
The particles of Ni_61.9_Al_38.1_ alloy. **a** Micron-sized particle, which has solidified from the molten droplet and **b** the foam from nanosized particles, which coats the surface of micron-sized particle

In the first glance, there is no great difference between the morphologies of the powders formed due to the quenching into the coolant liquid or condensation from the vapor state (Fig. 2a–c). However, two main evidence forces us to make the difference. Firstly, SEM studies reveal that solidified molten droplets usually appear on images as the separated spherical particles with a well distinguishing surface (Fig. 3a). In most cases, the foam-like shell that formed from the nanosized particles coats the surface of micron-sized particles (Fig. 3b). Secondly, there is a large difference between the particle size distributions of the micron-sized and nanosized particles. For the former, the maximum volume size distribution is located near 25–50 μm dependently on the alloy compositions and parameters of spark erosion processing while for the latter, most of the particles have sizes less than several tens nanometers. At the same time, the particle sizes of several hundreds nanometers arise sporadically. These facts allow us to suppose that the different mechanisms are responsible for the formation of micron- and nanosized particles.

### Nanosized Particles

**Fig. 4:**
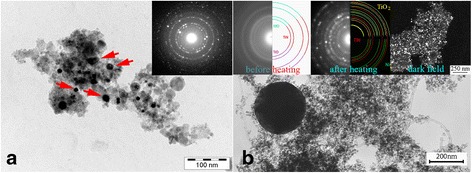
Nanosized particles obtained in liquid nitrogen. **a** Ti (on inset diffraction of δ-TiN); *arrows* indicate the cuboid particle. **b** Ni_49_Cu_1_Ti_47_Zr_3_ nanopowder. DF image from the agglomerate and diffractions before and after heating in the column of microscope are shown in the *insets*

**Fig. 5:**
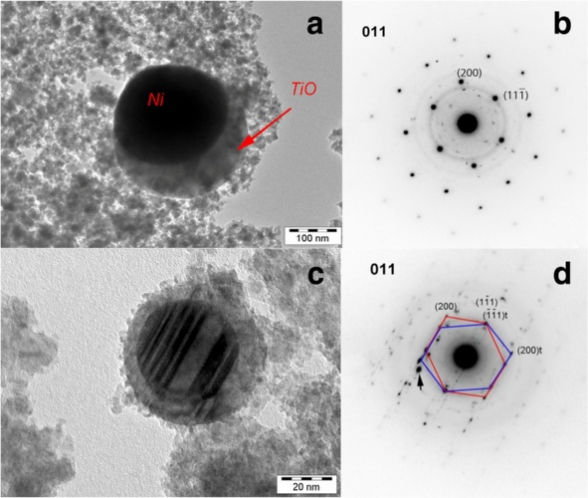
Composite particles in Ni_49.9_Ti_40.3_Zr_0.3_Cu_0.1_Hf_9.4_ powder. **a** The core is Ni, and the shell is TiO. **c** Twinned particles covered with oxide shell. *Spots* on the diffractions **b** and **d** correspond to the elemental Ni (zone axes: [*011*])

**Fig. 6:**
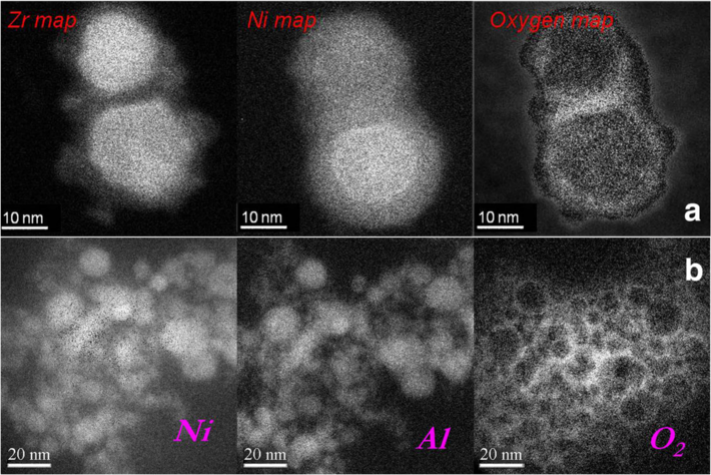
Element distributions in nanosized particles. **a** Zr_57_Cu_20_Al_10_Ni_8_Ti_5_ and **b** Ni_61.9_Al_38.1_ alloys obtained in liquid argon

**Fig. 7:**
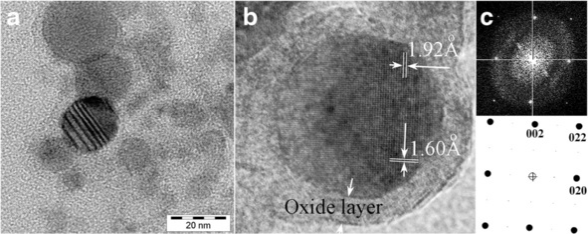
Structure of Ni-Al nanoparticles. **a** Striation contrast on BF low-magnification HRTEM image. **b** HRTEM image of the tetragonal lattice of L1_0_ martensite (incident beam||[100]_M_ direction) in the particle sized of 13 nm. **c** FFT power spectrum of the image (**b**) and its interpretation

**Fig. 8:**
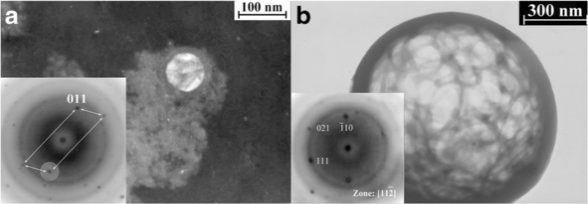
Particles of Ni_46.7_Ti_49.2_Zr_3.41_Cu_0.8_ powder in a martensitic state (B19′ structure). **a** Dark-field image (from (011) spot) of an 80-nm particle having a distinct internal structure and embedded in agglomerates of nickel and titanium oxides. **b** Bright-field image of the hollow particle with a diameter of 1 μm

TEM investigation revealed that the finest particles with a dimension down to 5 nm have a trend to form agglomerates (Figs. 4, 5, 6, 7, and 8). The prevailing particle morphology is spherical although sometimes it is very difficult to clearly recognize the shape of the smallest particles assembled in chains or agglomerates. In addition, in Ti powders obtained in liquid nitrogen, the cuboids were found here and there [27], which is typical for ultrafine δ-TiN_*x*_ particles [33] (Fig. 4). Most of the particles have size below 40 nm; however, the spherical particle sizes of ~100 ÷ 1000 nm appear on TEM images occasionally (Figs. 2b, 4b, 5a, 8a).

The electron diffraction taken from the agglomerates consists of diffuse rings related obviously to the finest particles and distinct spots related to the strictly crystalline structure of the largest particles (inset of Fig. 4). It is seen from the diffraction on the left inset of Fig. 4b that the finest particles of Ti-Ni-based alloys are rather the oxides of the components of alloys components than the alloys themselves. It is interesting that the NiO particles are reduced to elemental Ni, and simultaneously, TiO is oxidized to TiO_2_ after the heating of the nanosized particles of Ni_49_Cu_1_Ti_47_Zr_3_ and Ni_49.9_Ti_40.3_Zr_0.3_Cu_0.1_Hf_9.4_ alloys. The phenomenon was observed independently on the ways of the heating described in details elsewhere [31, 32].

Direct composition measurements by EDX and EFTEM methods of the particle size 10 ÷ 100 nm confirm that their compositions differ from the master alloys for the most alloys under consideration. In the case of the multicomponent Zr_57_Cu_20_Al_10_Ni_8_Ti_5_ alloy, the composition differs from particle to particle (Fig. 6a). In Ni_49_Cu_1_Ti_47_Zr_3_ and Ni_49.9_Ti_40.3_Zr_0.3_Cu_0.1_Hf_9.4_ alloys, the fine particles are mostly the oxides of the alloy components [31, 32]. At the same time, the element stratification is observed in the particles of the Ni_49.9_Ti_40.3_Zr_0.3_Cu_0.1_Hf_9.4_ alloys with a diameter of ~20 ÷ 150 nm (Fig. 5). The core of such particles is an elemental Ni, the shell is TiO oxide, and the averaged compositions of such particles are close to Ni_2_Ti [32]. In addition, the diffraction patterns taken from the cores of such particles correspond to the FCC structure of the elemental Ni. Sometimes, particles, especially those with moderate sizes (20 ÷ 30 nm), have a strictly twinned structure (Fig. 5c). Twinning in nickel cores is likely to appear as a reaction on the stresses from the oxide layer, which covered the Ni core from all sides.

Among the powders under consideration, only the nanosized particles of Ni_61.9_Al_38.1_ alloy have the composition close to the composition of the master alloy (Fig. 6b), which could be explained by the particular relations between the vapor pressures of Ni and Al elements. Indeed, the average composition measured by EFTEM method in separate Ni-Al particle sizes of 10 ÷ 50 nm was Ni66.3-Al37.7 at.% while the average composition of agglomerates is Ni59.2Al40.8 at.%. The difference is likely caused by the layer of aluminum oxide, which covers the core of the particle with the composition close to Ni_66.3_Al_37.7_. This assumption is confirmed indirectly by the HRTEM method, which shows that most of such particles demonstrated striated contrast typical for the twinned structure of martensite crystals. In addition, the observed interplanar distances as well as their relations and the observed angles on the fast Fourier transforms (FFT) power spectra were practically the same as for L1_0_ tetragonal structure of martensite, which is observed in bulk Ni-Al alloys with that composition. This situation drastically contrasts with the Ti-Ni-Hf cases, where striation contrast is caused by the twinning in the Ni core.

The discovery of the crystal structure of the particles of Ni_46.7_Ti_49.2_Zr_3.41_Cu_0.8_ alloy sized above ~100 ÷ 200 nm faced out the experimental difficulties. Those particles easily escaped from the field of view and/or carbon substrate due to any manipulation (tilting, rotation, focusing, etc.) needed to obtain good diffraction conditions, most of them were not transparent in addition. Nevertheless, in some cases one can say that such particles have a distinct crystal structure different from that of the smallest particles. Moreover, they are in a martensitic state.

In spite of the impossibility in obtaining perfect diffraction patterns, one can suppose that those of the group of reflexes, which form a certain part of a diffraction pattern, belong to the martensite (B19′ structure). Although identified correctly, zone axis was not possible (Fig. 8a) due to mentioned experimental reasons, and the observed interplanar distances were at least absolutely closed to martensitic distances in majority. Such particles usually had an internal structure revealed from selected area electron diffraction (SAED), which is very typical for martensite (Fig. 8a). The martensitic crystal structure of the relatively large particles indirectly confirms that such particles have the compositions close to the master alloy. Indeed, the martensite transformation in Ti-Ni-based alloys is possible only in a relatively narrow interval of Ni concentration near the quasi-equiatomic composition, with Zr and Hf replacing Ti and Cu replacing Ni [34].

A very interesting example of a particle with a size of 1 μm is shown on Fig. 8b. It seems to be a unique example when a diffraction pattern of acceptable quality could be indicated. Moreover, a so large particle (as for TEM studies) was transparent that clearly reveals the hollowness of the particle. The width of the walls observed as nontransparent circular border can be estimated as 50 nm.

### Mechanisms of the Nanoparticles Formation

At least two main mechanisms of the formation of nanosized particles of spark erosion powder are possible. The difference between the compositions of nanosized fraction of powders and master alloys is the distinct feature, which supposes the formation of nanosized particles through the condensation of vapor elements of alloys. Definitely, the finest nanosized particles (less then ~10 nm) appear as a result of the condensation of the vapor elements of the alloys. Its compositions depend on the vapor pressures of its constituents, the regimes of the spark erosion processing, and contaminants in the coolant liquid. Because of the contamination of the coolant by oxygen and followed by passivation of powder, those particles are the oxides of the components of the alloys gathered in the agglomerates rather than the metallic particles actually.

In turn, the presence of relatively large particles (up to ~200 nm) also with different compositions than that of the alloy, which appear as stand-alone particles or as embedded in agglomerate, allows us to assume that scenarios of condensation could be different for the finest particles and particles that are moderate in sizes. It is not a great overstatement to suppose that moderate-sized particles are formed during much more time—long enough that different constituents are condensed in one particle. Certainly, the compositions of such particles are defined by the concentrations of elements in vapor, i.e., the vapor pressures. Occasionally, these concentrations in Ni-Al are such that provide the composition of nanosized particles close to the master alloy while in Ti-Ni-based alloys, the concentration of Ni is more than that of Ti; Hf was absent as a whole in Ti-Ni-Hf-nanosized powder [32]. This observation corresponds to the conclusions made in [28] that Ni is evaporated from the surface of molten droplets more extensively than Ti, Zr, and especially Hf providing concentration deviation in the micron-sized particles.

**Fig. 9:**
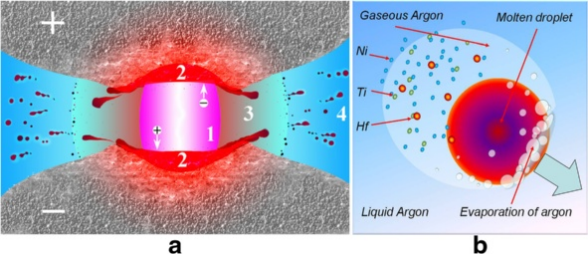
On the mechanism of particles formation during spark erosion. **a** The potential difference between positively and negatively charged granules of a material induces the arc discharge through the small gap between them. Arc plasma channel 1 produces a lot of heat which melts and evaporates the material. Around the channel, the gaseous bubble 3 appears, by surface of which those evaporated constituents are condensed. The jets of melt are ejected from the pulls of molten material 2 on the surface of cathode and anode. Liquid jets are subsequently transformed in micron-sized droplets, which are quenched in situ into the coolant liquid 4. **b** Illustration of quenching in situ the molten droplet, which is moving through the cryogenic liquid (argon) surrounded by the bubble of gaseous argon and vapors of alloy components (Ti, Ni, and Hf in that case)

The extensive evaporation of elements during spark erosion processing goes at once with two main processes. First, evaporation accompanies an electrical discharge from the molten pool on the surface of the pieces of the material followed by the condensation on the walls of a gaseous bubble near the arc plasma column (Fig. 9a). Second, an occasion happens during the cooling of the molten droplets moving through the coolant (Fig. 9b). While the duration of the first process is around 10–100 μs (till the bubble collapses), the duration of the evaporation period under cooling is much shorter. In that case, condensation goes on the walls of small bubbles, which are continuously coming off from the moving molten droplets and then collapsing. It seems this process is responsible for the smallest nanoparticles formation.

There is one more reason for the prolongation the vapor condensation in case of Ti-Ni-base alloys. The heat, which is realized during the formation of TiNi intermetallic compound, can slow down the cooling rate. Firstly, it provides further condensation of constituents, and secondly, the remelting of the agglomerates is composed of the earlier condensed particles even if such particles have already oxidized. In turn, strong affinity of Ti to oxygen provides the operation a kind of “oxygen pump”, which pulls out the oxygen atoms from the middles of such agglomerates to the surfaces simultaneously reducing the NiO oxide and producing TiO_2_ oxide. As a result, the Ni atoms tend to diffuse inside while Ti atoms to the surface of the particles. Thus, expansion of particles in size and the stratification of elements happen (Fig. 5).

At the same time, the particles, which have sizes above tens of nanometers, are solidified from the molten droplets thus reproducing the composition of the melt more or less for the most of the mentioned powders. One could expect that the amount of the submicron particles should be minor as it is in the case of the gas atomization. Indeed, the physical mechanism of the particles formation is very similar. It is the solidification of molten droplets formed due to the breaking of liquid jets, which are ejected from the molten pools of materials in case of spark erosion and sprayed from the nozzle in case of gas atomization. In both cases, the limitation factor is the surface tension, which restricts the particles size distribution from below. In both cases, the key role in the breaking mechanism plays the Rayleigh instability.

Another possible reason, which is responsible for the formation of the smallest particles in the case of spark erosion, can be explosive boiling of overheated molten jets followed by their explosion with formation submicron particles. This phenomenon can happen due to a rapid drop of pressure from 10^6^ ÷ 10^10^ Pa to atmospheric pressure [10, 35, 36] when the discharge is broken and the gaseous bubble around the arc column collapses. One of the results of such scenario is blowing-out the hollow particles from the molten droplets oversaturated by a coolant gas [to be published]. Such a micron-sized hollow particle is shown on Fig. 8, but much more number of the micron-sized hollow particles has been observed in [13–15, 30]. A drastic difference between the cryogen gas solubility in molten droplets and in the solid phase, which is assumed in [14, 15], promotes the explosive boiling mechanism. Evidently, in the case of an explosive breaking of the molten hollow particle, the sizes of the ejected particles will be comparable with the bubble wall width, namely several submicrons.

## Conclusions

Different morphology and composition of nanosized particles obtained in cryogenic liquids during the spark erosion processing of the pre-alloyed material supposes several mechanisms of their formation. One of them relates to the condensation of the evaporated constituents of alloy under consideration, and the finest particles (less then ~10 nm) are the oxides of the components of the alloy rather than alloy itself. In any case, the particles of all sizes are covered with the oxide layer. The observed composition of the condensed nanoparticles sized above than ~10 nm depends on (a) the relations between the vapor pressures of the constituents of the alloy, (b) duration of the condensation process, (c) alloy composition and affinity of the constituents to oxygen, and (d) the conditions of storage and manipulation with the powder. Therefore, the composition of such particles can be very close to the master alloy (Ni_66.3_Al_37.7_), which strongly differs from particles to particles for the multicomponent alloys (Zr_57_Cu_20_Al_10_Ni_8_Ti_5_) and in addition, can be stratified (Ti-Ni-based alloys). Another mechanism, which is responsible for the formation of particle sizes above several tens and even hundreds nanometers, ensures the coincidence of the composition of the nanoparticles with the master alloys. It supposes that the explosive breaking of the molten micron-sized bubbles or droplets ejected from the melt pools on the surface of the granules of the material.

## Abbreviations

BF: bright field (image)
DF: dark field (image)
EDX: energy-dispersive X-ray spectroscopy
EFTEM: energy-filtered transmission electron microscopy
FFT: fast Fourier transforms
HRTEM: high-resolution transmission electron microscopy
SAED: selected area electron diffraction
SEM: scanning electron microscopy
TEM: transmission electron microscopy
XRD: X-ray diffraction
